# A male case of an undifferentiated carcinoma with osteoclast-like giant cells originating in an indeterminate mucin-producing cystic neoplasm of the pancreas. A case report and review of the literature

**DOI:** 10.1186/1477-7819-9-100

**Published:** 2011-09-08

**Authors:** Takeyuki Wada, Osamu Itano, Go Oshima, Naokazu Chiba, Hideki Ishikawa, Yasumasa Koyama, Wenlin Du, Yuko Kitagawa

**Affiliations:** 1 Department of Surgery, Eiju General Hospital 2-23-16 Higashiueno Taitouku Tokyo 110-8645 Japan; 2 Department of Pathology, Keio University, School of Medicine, 35 Shinanomachi, Shinjuku-ku, Tokyo 160-8582, Japan; 3 Department of Surgery, Keio University, School of Medicine, 35 Shinanomachi, Shinjuku-ku, Tokyo 160-8582, Japan

**Keywords:** undifferentiated carcinoma with osteoclast-like giant cells, Mucin-producing, Mucinous, Cystic neoplasm, Pancreas

## Abstract

We report a rare male case of an undifferentiated carcinoma with osteoclast-like giant cells originating in an indeterminate mucin-producing cystic neoplasm of the pancreas. A 59-year-old Japanese man with diabetes visited our hospital, complaining of fullness in the upper abdomen. A laboratory analysis revealed anemia (Hemoglobin; 9.7 g/dl) and elevated C-reactive protein (3.01 mg/dl). Carbohydrate antigen 19-9 was 274 U/ml and Carcinoembryonic antigen was 29.6 ng/ml. A computed tomography scan of the abdomen revealed a 14-cm cystic mass in the upper left quadrant of the abdomen that appeared to originate from the pancreatic tail. The patient underwent distal pancreatectomy/splenectomy/total gastrectomy/cholecystectomy. The mass consisted of a multilocular cystic lesion. Microscopically, the cyst was lined by cuboidal or columnar epithelium, including mucinous epithelium. Sarcomatous mononuclear cells and multinucleated osteoclast-like giant cells were found in the stroma. Ovarian-type stroma was not seen. We made a diagnosis of osteoclast-like giant cell tumor originating in an indeterminate mucin-producing cystic neoplasm of the pancreas. All surgical margins were negative, however, two peripancreatic lymph nodes were positive. The patient recovered uneventfully. Two months after the operation, multiple metastases occurred in the liver. He died 4 months after the operation.

## Background

Undifferentiated carcinoma (UC) with osteoclast-like giant cells (OGCs) is rare neoplasm of the pancreas. The tumor was first described by Rosai in 1968 [1], and similar tumors also have been identified in the skin, thyroid gland, ovary, breast, kidney, prostate, and soft tissue. In the pancreas, it was mostly recorded in ductal adenocarcinomas. Since Posen et al. reported the first case of an UC with OGCs of the pancreas associated with a mucus-secreting cystadenocarcinoma in 1981 [2], there have been 11 additional cases of UC with OGCs of the pancreas originating in mucinous cystic neoplasms (MCN) and indeterminate mucin-producing cystic neoplasm reported in the English language literature [2-12]. Among these cases, only one male case has been reported [8]. In this report, we describe a new male case of UC with OGCs that originated in an indeterminate mucin-producing cystic neoplasm of the pancreas, and discuss the clinicopathological features as well as present a review of the pertinent literature.

## Case report

**Figure 1:**
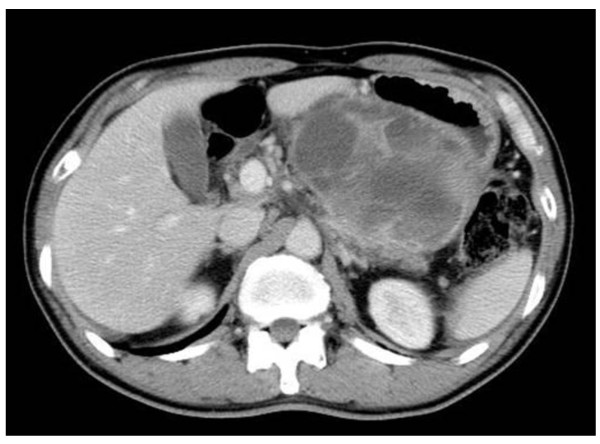
**Abdominal CT showing the large cystic tumor in the upper left quadrant of the abdomen**. A computed tomography scan of the abdomen revealed a large cystic mass appeared to originate from the pancreatic tail.

**Figure 2:**
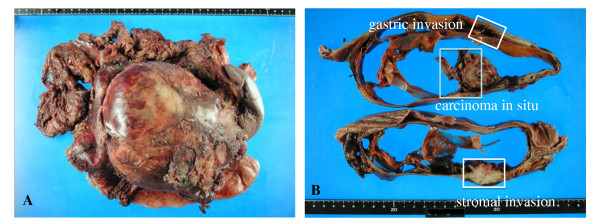
**Macroscopic findings showing a multilocular cystic tumor**. (A) A multilocular cystic tumor that was 20 cm wide at its largest diameter was located in the cauda pancreatis. (B) The cystic cavities, which were separated by thin, transparent septations, were filled with fluid of low viscosity. The pathological mapping shows carcinoma in situ, stromal invasion and gastric invasion.

**Figure 3:**
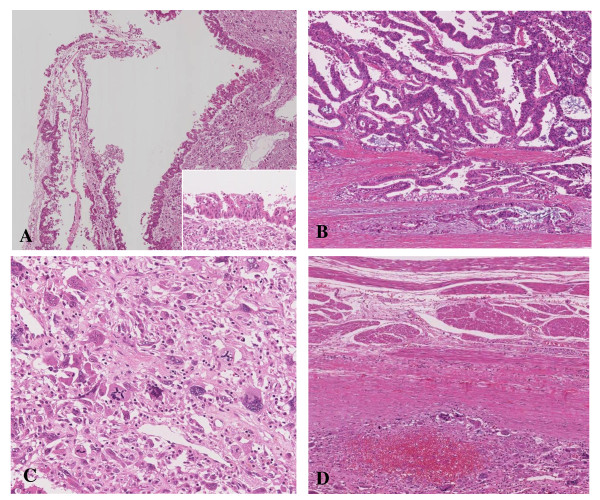
**HE staining image of the tumor tissue**. (A) The cystic spaces were lined by a columnar mucinous epithelium that presented papillary folding. Higher power view of columnar mucinous epithelium is displayed on the bottom-right corner. (B) There was a small number of stromal invasive features in the bottom of the solid part of this cystic tumor. (C) Near the carcinoma in situ, OGCs were distributed diffusely in the stroma of the cyst wall. (D) The tumor showed the invasion to the stomach across the serosal layer.

**Figure 4:**
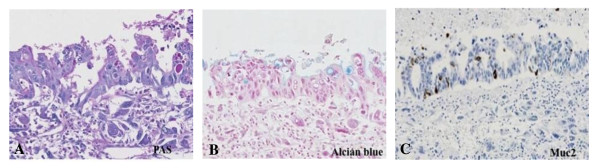
**Histological findings showing mucus production of cyst wall**. The epithelium of the cyst wall showed mucus production, as demonstrated by the positive reactions with PAS, alcian blue and Muc-2.

**Figure 5:**
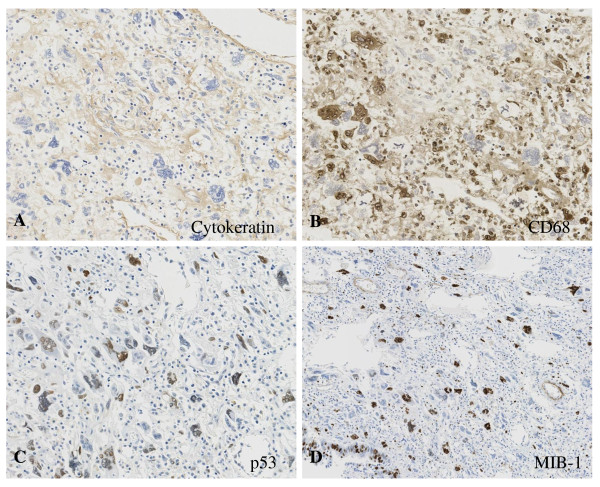
**Immunohistochemical examination of OGC and PLC**. (A) The stroma associated with OGCs and PLCs was negative for cytokeratin AE1/AE3. (B) OGCs expressed the histiocytic marker CD68. (C) Almost all of the PLCs were positive for p53. (D) The Ki-67 positivity of the stroma associated with OGCs and PLCs was about 30%.

A 59 year-old man presented at our hospital with a complaint of fullness in the upper abdomen. A physical examination showed a palpable mass in the upper left abdomen. Laboratory tests showed anemia and inflammatory reactivity, hemoglobin (Hgb) was 9.7 g/dl and C-reactive protein (CRP) was 3.01 mg/dl. Carbohydrate antigen 19-9 (CA19-9) was 274 U/ml and carcinoembryonic antigen (CEA) was 29.6 ng/ml. A computed tomography scan revealed a large cystic mass in the upper left quadrant of the abdomen that appeared to originate from the pancreatic tail (Figure 1). In magnetic resonance images, the cystic component showed variable signal intensities, and nodular components were seen in the cystic wall. Magnetic resonance cholangio-pancreatography showed narrowing and irregularity of the main pancreatic duct. Although it was a male case, we concluded tentatively that tumor might be a MCN of pancreas based on its characteristic appearance resembling the shape of an orange. An operation was performed. At laparotomy, a large cystic mass was found in the pancreas tail. The tumor invaded to the stomach, but distant metastasis was not discovered. The patient underwent distal pancreatectomy with splenectomy, total gastrectomy and cholecystectomy. Histological analysis revealed a multilocular cystic tumor that was 20 cm wide at its largest diameter and located in the cauda pancreatis (Figure 2-A). The cystic cavities, which were separated by thin, transparent septations, were filled with fluid of a low viscosity (Figure 2-B). In some parts the lining was dotted, occasionally presenting papillary projections. A 3-cm solid part was observed consisting of yellow to brown material. The cystic spaces were lined by a columnar mucinous epithelium that presented with papillary folding (Figure 3-A). The epithelium presented severe dysplasia, reaching the degree of a carcinoma in situ. The walls of the cysts did not display an ovarian-type stroma. There were a small number of stromal invasive features in the bottom of the solid part of this cystic tumor (Figure 3-B). Close to the carcinoma in situ, the OGCs were distributed diffusely in the stroma of the cyst wall, with more than 10 nuclei per cell and lacking features of atypia. In Figure 2-B we present views of the cut surface of the cystic tumor delineating the pathological mapping of carcinoma in situ, stromal invasion and gastric invasion. In the stroma of the cyst wall, some pleomorphic large cells (PLCs) were also observed. The PLC was a large cell with irregular, pleomorphic or bizarre nuclei and frequently demonstrating atypical mitosis (Figure 3-C). The tumor showed invasion to the stomach across the serosal layer (Figure 3-D). The epithelium of the cyst wall showed mucus production, as demonstrated by positive reactions with Periodic acid-Schiff stain (PAS), alcian blue and Muc-2 (Figure 4-A, B, C). The papillary epithelium was positive for the epithelial marker cytokeratin AE1/AE3, but the stroma associated with OGCs and PLCs was negative for cytokeratin AE1/AE3 (Figure 5-A). OGCs expressed the histiocytic marker CD68 (Figure 5-B). Almost all of the PLCs were positive for p53 (Figure 5-C) and negative for CD68. The Ki-67 positivity of the stroma associated with OGCs and PLCs was about 30% (Figure 5-D). This tumor was not diagnosed as a MCN, because it did not display an ovarian-type stroma. However, it seemed inappropriate to diagnose this tumor as an intra-ductal papillary mucinous neoplasm (IPMN), considering invasive features to stroma and stomach and lymph nodes metastases of this tumor. Therefore, we diagnosed our case as an indeterminate mucin-producing cystic neoplasm, according to the international consensus guidelines for management of intraductal papillary mucinous neoplasms and mucinous cystic neoplasms of the pancreas, in which an ovarian-type stroma is a histological requirement for the diagnosis of a MCN [13]. Based on these findings, this case was diagnosed as an UC with OGCs originating in an indeterminate mucin-producing cystic neoplasm of the pancreas. The patient recovered uneventfully and was discharged from the hospital on the 23rd post-operative day. Multiple liver metastases were detected 2 months after the operation, and the patient died 4 months after the operation.

## Discussion

Since Posen et al. reported the first case of an UC with OGCs of the pancreas associated with a mucus-secreting cystadenocarcinoma in 1981 [2], there have been 11 additional cases reported in the English language literature of UC with OGCs of the pancreas originating in MCN and indeterminate mucin-producing cystic neoplasm [2-12]. Only one male case was reported in addition to our case. We searched the literature by the PubMed database. The characteristics of our case and the previously reported cases are summarized in Table 1.

**Table 1:** Clinicopathological findings of UC with OGCs of the pancreas originating in mucinous cystic neoplasms (MCN) and indeterminate mucin-producing cystic neoplasm

Case	Author	Year	Age (years)	Sex	Location	Size (cm)	Symptom	Lymph node metastasis	Invasion to another organ	Ovarian-type stroma	Survival
1	Posen et al. [2]	1981	45	F	Body	14	Abdominal pain	-	-	ND	ND
2	Aoki et al. [3]	1989	44	F	Tail	15	Palpable tumor in the abdomen	-	-	ND	NR at 3 years
3	Bergman et al. [4]	1995	77	F	Head	5	Nausea, weight loss	-	-	+	Lost to follow up
4	Suda et al. [6]	2001	35	F	Tail	11	ND	+	-	+	NR at 14 years
5	Leighton et al. [5]	2001	40	F	Body&Tail	15	Back pain, nausea	-	-	ND	NR at 10 months
6	Sarnaik et al. [7]	2003	25	F	Tail	17	Abdominal pain	-	-	ND	NR at 22 months
7	Sedivy et al. [9]	2005	44	F	Tail	12	Anemia	-	-	+	NR at 48 months
8	Nai et al. [8]	2005	69	M	Head	5	Weight loss, jaundice	-	-	ND	Died at 1 year
9	Pan et al. [10]	2007	70	F	Body&Tail	14	Anemia, weight loss, appetite loss	-	-	+	NR at 4 months
10	Hirano et al. [11]	2008	26	F	Body&Tail	11	Abdominal pain	-	-	+	NR at 8 months
11	Burkadze et al. [12]	2009	34	F	Tail	11	Abdominal pain	-	-	+	NR at 4 years
12	Our case	2010	59	M	Tail	20	Fullness in the lower abdomen	+	+	-	Died at 4 months

ND, not described; NR, no recurrence

The reports described 2 men and 10 women ranging in age from 25 to 77 years with a median age of 47 years, suggesting that this type of tumor tends to develop in middle age and predominantly in females. That spectrum was compatible with that of ordinary MCN. Patients showed symptoms such as abdominal pain or discomfort, anemia, and weight loss. The tumor arose from the head of the pancreas in 2, body in 1, tail in 6, and body and tail in 3 patients. The lesions were resected in all of the patients. The average tumor size was 12.5 cm at the largest diameter, ranging from 5 to 20 cm. Lymph node metastasis was seen in two cases. Invasion to another organ was seen only in our case, in which the tumor invaded to the stomach. With the exception of the two male cases, the patients had experienced favorable courses of their disease and were alive when papers were published. An ovarian-type stroma was seen in 6 cases, and 5 cases did not mention it. Our case did not display an ovarian-type stroma.

Although some authors have stated that UC with OGCs of the pancreas is apt to present as a large mass with a slow metastatic spread and a much better prognosis than ordinary carcinoma [14,15], the prognosis of UC with OGCs of the pancreas originating in a MCN and indeterminate mucin-producing cystic neoplasm remains unclear due to the small number of reported cases and short follow-up periods.

Zamboni et al. reported that 14% of MCNs of the pancreas did not demonstrate an ovarian-type stroma and that these tumors had a high tendency to invade compared to the tumors with ovarian-type stroma [16]. Furthermore, some have suggested that MCN may lose its ovarian-type stroma with malignant transformation [17,18]. Our case did not display an ovarian-type stroma, and demonstrated gastric invasion and lymph nodes metastasis consisted of ductal adenocarcinoma component. And, similar to our case, another male case reported by Nai et al. [8] also died from liver metastasis 1 year after the operation. These authors did not state whether or not an ovarian-type stroma was present. An UC with OGCs originating in an indeterminate mucin-producing cystic neoplasm of the pancreas may also have a poor prognosis compared to an UC with OGCs originating in a MCN with ovarian-type stroma.

UC with OGCs is a rare neoplasm of the pancreas. In most cases, UCs with OGCs originate in ductal adenocarcinoma, classified as a subtype of undifferentiated carcinoma in the WHO classification [19], and are only rarely combined with MCNs. Since the first description of UC with OGCs by Rosai, the origin of the tumor has been controversial. In our case, OGCs were positive for the histiocytic marker CD68 and negative for p53. On the other hand, almost all of the PLCs were positive for p53 and negative for CD68. In this type of tumor, PLC may have a neoplastic potential and produce chemotactic and growth factors that stimulate the proliferation of circulating precursor cells to OGCs.

## Conclusions

In conclusion, we have reported a male case of UC with OGCs originating in an indeterminate mucin-producing cystic neoplasm of the pancreas. Because the number of cases is too small to arrive at definitive conclusions, more studies are needed to establish the treatment strategy for this tumor.

## Consent

Written informed consent was obtained from the patient for publication of this case report and any accompanying images. A copy of the written consent is available for review by the Editor-in-Chief of this journal.

## List of abbreviations used

UC: undifferentiated carcinoma; OGC: Osteoclast-like giant cell; MCN: Mucinous cystic neoplasms; Hgb: Hemoglobin; CRP: C-reactive protein; CA19-9: Carcinoembryonic antigen; PLC: Pleomorphic large cell; PAS: Periodic acid-Schiff stain; IPMN: Intra- ductal papillary-mucinous neoplasms.

## Conflict of interests statement

The authors declare that they have no competing interests.

## Authors' contributions

TW and OI wrote the manuscript. OI have operated this case. TW, GO, NC, HI and YK did the assistant of the operation. WD diagnosed the pathology of this case. YK reviewed the manuscript. All authors read and approved the final manuscript.
